# Formoterol dynamically alters endocannabinoid tone in the periaqueductal gray inducing headache

**DOI:** 10.1186/s10194-024-01907-y

**Published:** 2024-11-19

**Authors:** Ingrid L. Peterson, Erika Liktor-Busa, Kelly L. Karlage, Sally J. Young, Natalie E. Scholpa, Rick G. Schnellmann, Tally M. Largent-Milnes

**Affiliations:** 1 Department of Pharmacology, College of Medicine, University of Arizona, Tucson, AZ United States; 2 Department of Pharmacology and Toxicology, College of Pharmacy, University of Arizona, Tucson, AZ United States; 3 Southern Arizona VA Health Care System, Tucson, AZ United States; 4 Southwest Environmental Health Science Center, University of Arizona, Tucson, AZ United States; 5 Department of Neuroscience, College of Medicine, University of Arizona, Tucson, AZ United States; 6 Center for Innovation in Brain Science, University of Arizona, Tucson, AZ United States

**Keywords:** Headache, Endocannabinoid, Adrenergic, PAG, In vivo, Formoterol, Mouse

## Abstract

**Background:**

Headache is a pain disorder present in populations world-wide with a higher incidence in females. Specifically, the incidences of medication overuse headache (MOH) have increased worldwide. Comorbidities of MOH include photosensitivity, anxiety, “brain fog”, and decreased physical activity. The FDA-approved long-lasting selective β_2_-adrenergic receptor agonist, formoterol, is currently approved for use in severe asthma and chronic obstructive pulmonary disease. Recently, interest in repurposing formoterol for use in other disorders including Alzheimer’s disease, and neuropathic pain after spinal cord injury and traumatic brain injury has gained traction. Thus, revisiting known side-effects of formoterol, like headache and anxiety, could inform treatment paradigms. The endocannabinoid (eCB) system is implicated in the etiology of preclinical headache, with observed decreases in the circulating levels of endogenous cannabinoids, referred to as Clinical Endocannabinoid Deficiency. As cross-talk between the eCB system and adrenergic receptors has been reported, this study investigated the role of the eCB system and ability of formoterol to induce headache-like periorbital allodynic behavior.

**Methods:**

Female 8-week-old C57Bl/6J mice were treated daily with formoterol (0.3 mg/kg, i.p.) for up to 42-days, during which they were assessed for periorbital allodynia, open field/novel object recognition, and photosensitivity. At the end of the study, the periaqueductal grey (PAG), a brain region known to contribute to both headache induction and maintenance, was collected and subjected to LC-MS to quantify endocannabinoid levels.

**Results:**

Mice exhibited periorbital allodynia at nearly all time points tested and photosensitivity from 28-days onward. Levels of endocannabinoids, anandamide (AEA) and 2-arachidonoylglycerol (2-AG), along with cannabinoid receptor 1 (CB_1_R) expression were altered by both age and upon treatment with formoterol. Administration of FAAH/MAGL inhibitors, to target the eCB system, and a non-selective cannabinoid receptor agonist, WIN 55,212 reversed the formoterol-induced periorbital allodynia.

**Conclusions:**

These results suggest that formoterol is dysregulates eCB tone to drive headache-like periorbital allodynic behaviors. These results could help inform preventative treatment options for individuals receiving formoterol, as well as provide information on the interaction between the eCB and adrenergic system.

**Graphical Abstract:**

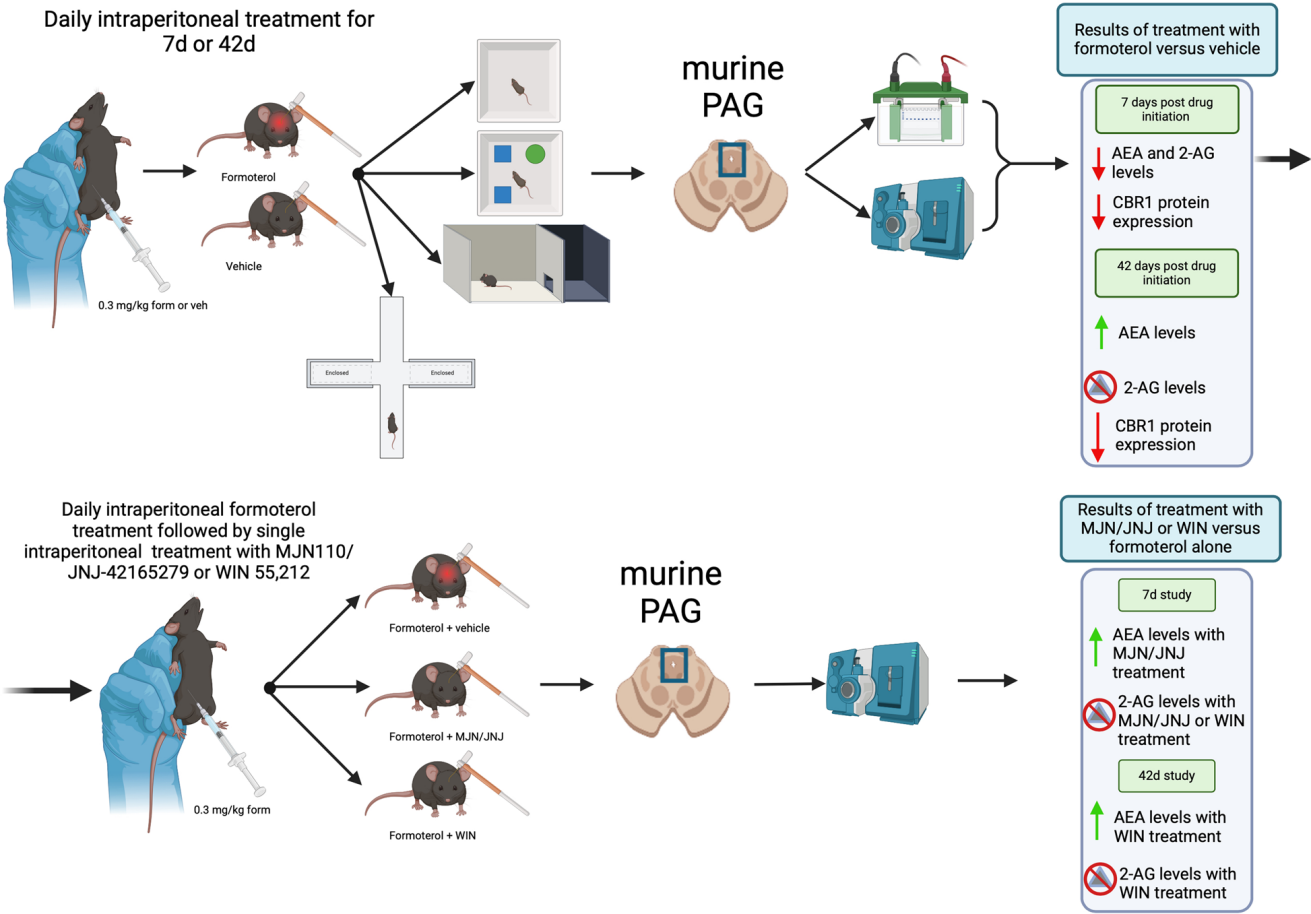

**Supplementary Information:**

The online version contains supplementary material available at 10.1186/s10194-024-01907-y.

## Background

Headache is a debilitating disorder experienced world-wide in both sexes with a prevalence that continues to increase [1–3]. This broad classifier is made up by numerous different types of headache disorders ranging from migraine to medication-overuse headaches (MOH) [1]. There are an estimated 45 million people experiencing un-specified “headache”, with a disproportionate impact on the female population [4]. Migraine sufferers account for 39 million of these individuals, with this primary headache disorder also being considered a painful and debilitating condition that can alter the daily life of a patient [4, 5]. Medication-overuse headache (MOH) falls into the secondary headache disorder category [6] and is a known contributor to the transition from episodic to chronic headache disorders [7]. This type of headache is considered a secondary chronic headache disorder occurring in about 63 million people worldwide, though due to underreporting this number may be greater [8, 9].

Several comorbidities have been observed in headache/migraine patients, such as anxiety, depression and photophobia [10, 11]. A bidirectional relationship between anxiety and depression has been observed in migraine patients, where the presence of one is often correlated to the development/occurrence of another [10]. While photophobia can be experienced due to other factors such as dry eye disease, optic nerve damage, traumatic brain injury, etc. [11], this condition occurs more frequently in those that experience headaches (40% of sufferers) than the general population [12]. Photophobia can manifest in such ways as an increase in headache intensity, discomfort, and ocular pain [12].

Although the underlying pathology of headache remains only partially understood, endogenous differences in neuromodulators in pain networks, for example the endocannabinoid (eCB) system, are hypothesized to be of importance [13]. In vertebrates, this system is known to have important roles in development of the nervous system, regulation of the endocrine and immune systems, modulation of neuronal activity and network function in the mature nervous system, and energy balance [14–17]. There are multiple different aspects of this signaling system, this includes endogenous ligands 2-arachidonoylglycerol (2-AG) and anandamide (AEA) and enzymes monoacylglycerol lipase (MAGL) and fatty acid amide hydrolase (FAAH) that serve as principal catabolic enzymes, as well as cannabinoid receptors 1 and 2 (CB_1_R and CB_2_R) [5, 18–20]. Additionally, dysregulation of this system has been implicated in some overlapping symptomologies with headache, such as learning and memory processes, and the development of anxiety [21, 22]. The eCB system is also hypothesized to alter morphology and respiratory function of mitochondria within brain tissue, modulate the release of neuropeptides, which play crucial roles in migraine, alter nitric oxide synthesis and neurovascular tone, and impact synaptic transmission [5, 23, 24]. Further supporting the role the eCB system plays an important role in the prevalence of headache is the occurrence of Clinical Endocannabinoid Deficiency, which are chronically low levels of 2-AG and AEA in the platelets and cerebral spinal fluid of migraine patients [13]. Preclinical models of cortical spreading depression opioid- and sumatriptan-induced headache (MOHs), and acute inhibition of DAGLA showed reduced 2-AG in the periaqueductal gray (PAG) at time points associated with periorbital allodynia [25, 26]. AEA levels were elevated, decreased, and unchanged in the cortex in the CSD, DAGLα, and MOH models, respectively; PAG levels of AEA were not changed. Endocannabinoid levels in the trigeminal nucleus caudalis (Vc) and trigeminal ganglia (TG) were not altered in these headache models either [25, 26]. Additionally, studies in rodent models in which MAGL and FAAH were inhibited showed an increase in levels of 2-AG and AEA and a decrease in headache-like behaviors [27, 28]. While eCB system receptors are found throughout the body, the most prevalent eCB receptor within the central nervous system is CB_1_R [18]. Regions implicated in headache/migraine pain generation that contain localized concentrations of CB_1_ receptors include, but are not limited to, the PAG, Vc, and TG [5, 13].

The FDA-approved drug, formoterol, is a long-lasting selective β_2_-adrenergic receptor (ADRB2) agonist that works as a bronchodilator and is prescribed as an inhalant for use in patients with chronic obstructive pulmonary disease and severe asthma [29, 30]. This drug has been shown to cross the blood brain barrier (BBB) [31, 32] with it being investigated for use in a variety of pain processes and models of neuropathic pain, including but not limited to sciatic nerve cuffing [33, 34], spinal cord injury (SCI) [35, 36], paclitaxel-induced pain [37], and spared nerve injury [38]. There has also been interest in repurposing the drug for a variety of addition disease processes including the treatment of SCI [39], acute kidney disease [40], diabetic neuropathy [40] and muscle atrophy/wasting [39, 41]. Unfortunately, however, human patient data has reported adverse effects of formoterol treatment, including headache, dyspnea, nasopharyngitis, and pharyngitis [42, 43]. Notably, the mechanism of formoterol induced headache pain has not yet been elucidated [42, 43].

The noradrenergic system, which contains the receptor target of formoterol, is intimately involved in modulation of the emotional state—such as anxiety and stress—and the immune system [38]. Studies investigating the role of the eCB system in modulation of emotional homeostasis and anxiety suggest an important interaction with the noradrenergic receptors [44]. In a rodent model of memory, α_2_-adrenergic receptors were shown to be involved in context-dependent fear memory and impairment [44]. Additionally, when metabolism of AEA is blocked using the fatty acid amide hydrolase inhibitor URB597, there was an observed decrease in the levels of ADRB2 within the hippocampus of female mice—this suggests that, in chronically stressed animals, altering levels of AEA and ADRB2 has a sex-specific impact on long-term memory [45]. Additionally, when WIN55,212, a synthetic cannabinoid agonist, was given to rats for both short and long term time points there were significant alterations observed in levels of adrenergic receptors α_2_ and β_1_ in the frontal cortex [46]. With the known interactions between the noradrenergic system and the eCB system in these processes, it is possible that a connection exists underlying the mechanism of formoterol-induced headache-like pain.

Given the existing data describing the consistent plasticity of the eCB system within the PAG across preclinical headache models [25], as well as the reported crosstalk between it and the adrenergic signaling system in other systems [14, 47–49], this study aimed to examine the interaction of formoterol with the eCB system and its effect on endocannabinoid tone, and how this contributes to headache-like behavior and molecular changes within the PAG.

## Methods

### Animals

Female wild-type C57Bl/6J mice 7–8 weeks of age were sourced from The Jackson Laboratories (Bar Harbor, ME) and housed in groups of 3–4. Upon arrival, mice acclimated for 3 days prior to introduction to behavior rooms and behavioral-assay acclimatization. Because headache disproportionately impacts the female population, the studies described below were conducted in female mice. For all behavioral assays, after the initial acclimation period in the animal facility, mice were then acclimated to their respective behavior rooms for 30-minutes and then placed in the behavior apparatus as dictated by the assay being conducted. Age-matched naïve mice were utilized for some assays, denoted below.

All animal work and studies presented were approved by the Institutional Animal Care and Use Committee of the University of Arizona (Approval 17–223) in accordance with the guidelines set forth by the National Institutes of Health Guide for the Care and Use of Laboratory Animals and the International Association for the Study of Pain.

### Harvest of tissue samples

Mice were euthanized using 5% isoflurane in 100% O_2_ at 2L/min followed by transcardial perfusion using 20mL of pre-chilled, ice-cold 0.1 M phosphate buffer, followed by decapitation. Brain regions involved in headache pain signaling were collected, flash frozen in liquid nitrogen or dry ice, and stored at -80°C until molecular assay preparation. The whole PAG was collected then hemisected into left and right. For consistency, the left side of the collected tissue was used for liquid-chromatography mass-spectrometry and the right side was used for western blotting (WB).

### Drug treatment

Formoterol fumarate dihydrate was sourced from Sigma-Aldrich (St. Louis, MO), dissolved in DMSO to 10mg/mL and aliquoted into 30µL amounts, which were then brought up in 10mL of saline solution to a final concentration of 0.03 mg/mL with < 1%DMSO. Mice were treated daily via intraperitoneal (i.p.) injection with 0.3 mg/kg formoterol or vehicle (DMSO + saline). Drug administration began a time-point zero and continued daily until the end of the study.

Tween80 and saline solution, MJN110 (Cayman #17583), JNJ-42165279 (Cayman #19987), and WIN55,212 (Cayman #10736) were brought up to a working concentration in a 10:10:80 ratio of DMSO, Tween80 and saline via i.p., 200 µL as follows: MJN110/JNJ-42165279 10mg/kg, WIN55,212 1mg/kg [26, 50, 51]. The MJN/JNJ combination drug—mixed into a single injectable—serve as inhibitors for the enzymes MAGL and FAAH, and the WIN treatment as a cannabinoid receptor targeting agent.

### Assessment of periorbital allodynia

Von Frey filaments were used to assess periorbital allodynia [52]; mice were acclimated to the assay room for 30-minutes and to the apparatus for 1-hour prior to measurements. Measurements were taken in a room at 354 ± 10 lux. Animals were exposed to the monofilaments and tester for 7 days prior to baseline measurements, where they could approach, smell, and investigate the filaments and the hand of the tester freely during this time. The monofilaments used were: 2.36 (0.02 g), 3.61 (0.4 g), 3.84 (0.6 g), 4.08 (1 g), 4.17 (1.4 g), 4.31 (~ 2 g). The experimenter was consistent and blinded throughout the studies. Monofilaments were applied to the periorbital area of the mice until either a reaction occurred, or 3-seconds had lapsed; measurements began with the 4.08 (1 g) monofilament and were used in line with the “up-down” method. Measurements were taken until 4 measurements were taken after the first positive withdrawal or the minimum or maximum monofilament strength was used. Baseline measurements were taken prior to drug administration, behavior assessment began 7-days post initiation and continued weekly up to 42-days.

For the inhibitor study, a separate cohort of mice underwent the pre-drug baseline, after which the entire cohort received formoterol daily for either 7 or 42 days. On the final day of each study, a post-drug periorbital von Frey baseline was conducted and then mice were given either vehicle, the MJN/JNJ inhibitor cocktail or WIN alone. Von Frey measurements were then taken every 30-minutes after administration for 4 hours in a blinded fashion.

### Assessment of photophobia

Sensitivity to light was measured using light/dark boxes (PanLab, Harvard Apparatus) at 6 variable lux ± 10 lux: 23.8, 53.1, 107.7, 163, 300.42, and 735. These luxes range from approximately equivalent to twilight, up to traditional office lighting and a cloudy day. Mice were acclimated to the assay room and to the chambers for 30-minutes under 107.7 lux two separate times, after which baseline measurements were taken. Measurements were taken at 4- and 7-days, and weekly up to 42-days after drug initiation in age-matched naïve and drug (formoterol or vehicle) treated mice in a blinded fashion. Data was collected using PPCWin. Time in light aversion index was calculated using the following formula [53]:

$$\:Aversion\:index=\frac{\left({Time\:in\:light}_{BL}\right)-\left({Time\:in\:light}_{test}\right)}{{Time\:in\:light}_{BL}}$$

### Assessment of anxiety-like behaviors: Elevated Plus Maze

Using the elevated-plus maze apparatus (PanLab, Harvard Apparatus), anxiety-like behaviors were assessed at baseline 4 and then 7 days, and weekly up to 42-days post-drug initiation. Mice were acclimated to the assay room; tests were conducted for 5-minutes/test and measurements were collected using AnyMaze software.

### Quantification of 2-AG and AEA by LC-MS

Age-matched naive mice were used throughout this study, as levels of AEA and 2-AG have been observed to vary naturally with age [54–56]. Tissues were harvested, snap frozen and stored at -80°C until use. On assay day, tissue weights were obtained and PAG samples for LC-MS were purified by organic solvent extraction on ice [13, 57] using 1mL of chloroform-methanol (2:1) supplemented with 1mM of PMSF per sample to inhibit degradation via endogenous enzymes during the tissue preparation processes. Tissue was then homogenized using mechanical sonication four times for 10-seconds per round on ice. After homogenization, 300µL of 0.7% NaCl was added to samples, followed by vortexing for 10-seconds and centrifugation for 10-minutes at 3200g at 4°C. The organic phase was then transferred to a new glass vial. 800µL of chloroform was added to the remaining aqueous phase, vortexed and centrifuged as described above; organic phases were pooled together. The extraction process was performed once more for a total of 3 rounds, after which the remaining aqueous phase was discarded. Once complete, 6µL of glycerol-methanol (3:7) solution was added to the pooled organic phases and samples were placed on an inert gas evaporator for 45-minutes or until completely evaporated. Once evaporated, the resulting products were redissolved in 200µL of chloroform, collected into a new vial and, to precipitate proteins, 1mL of ice-cold acetone was added. Samples were then vortexed and centrifuged for 5-minutes at 1800g at 4°C. The resulting organic phases were pooled into new glass vials and 6µL of glycerol-methanol (3:7) was added to each sample, followed by the following internal standards: 2AG-d5 (Cayman 362162) at 100µg/200µL and AEA-d4 (Cayman 10011178) at 100µg/100µL. Standard make up and analysis of 2-AG and AEA was performed as described in Levine et al. 2021 [13].

### Immunoblotting

Flash-frozen tissue was processed on ice using 300µL Tris-HCl based lysis buffer combined with 100x Halt protease/phosphatase inhibitor cocktail (Thermo #87786) for protein isolation. Tissue was homogenized via mechanical sonication using three 5-second pulses, after which samples were rocked at 4°C for 30-minutes for maximum combination of homogenized tissue with lysis buffer. After rocking, samples underwent centrifugation for 10-minutes at 12,000x rpm at 4°C. To measure protein content, the Pierce BCA Protein Assay Kit (ThermoScientific 23225) was used. Sample lysates were made up to a final concentration of 20 µg of protein per 16µL/sample, which was then separated using electrophoresis using 4–20% gradient gels (BioRad #5671093) and transferred to nitrocellulose membranes (Fisher #45-004-001). The resulting membranes were blocked in 5% milk made in 1% TBS for 30-minutes. Primary antibodies were made up in 5% BSA in 1% TBST, applied to the membranes, and incubated for 48-hours at 4°C with constant agitation. Membranes were then washed and incubated with fluorescently tagged secondary antibodies for 1-hour and imaged using a Sapphire Biomolecular Imager (Azure Biosystems, Dublin, CA). Housekeeping proteins were probed for on the same gel as each target protein to assess consistent loading. Primary antibodies used were as follows: Recombinant Anti-Cannabinoid Receptor I antibody (1:1000, Abcam), Anti-beta Actin antibody (1:10,000, Abcam), Rabbit monoclonal [EPR707(N)] to β_2_ Adrenergic Receptor (1:2,000, Abcam). Densitometry was measured using the UN-SCAN-IT software (Silk Scientific Inc, UT).

### Statistical analysis

The behavior of a single animal to tissue isolated from a single animal represents an *n* = 1.

GraphPad Prism 10.0 software (GraphPad Software) was used for statistical analysis. Unless otherwise noted, data are expressed as mean ± SEM. To determine numbers needed for each experiment G.Power3.1 was used for 80% power to detect a 20% difference when alpha = 0.05. Normality was tested and groups were compared by unpaired t-test or one-way ANOVA or two-way ANOVA with Tukey’s post-test, as indicated. Differences were considered significant if p ≤ 0.05.

## Results

### Dosing with formoterol induces periorbital allodynia

**Fig. 1:**
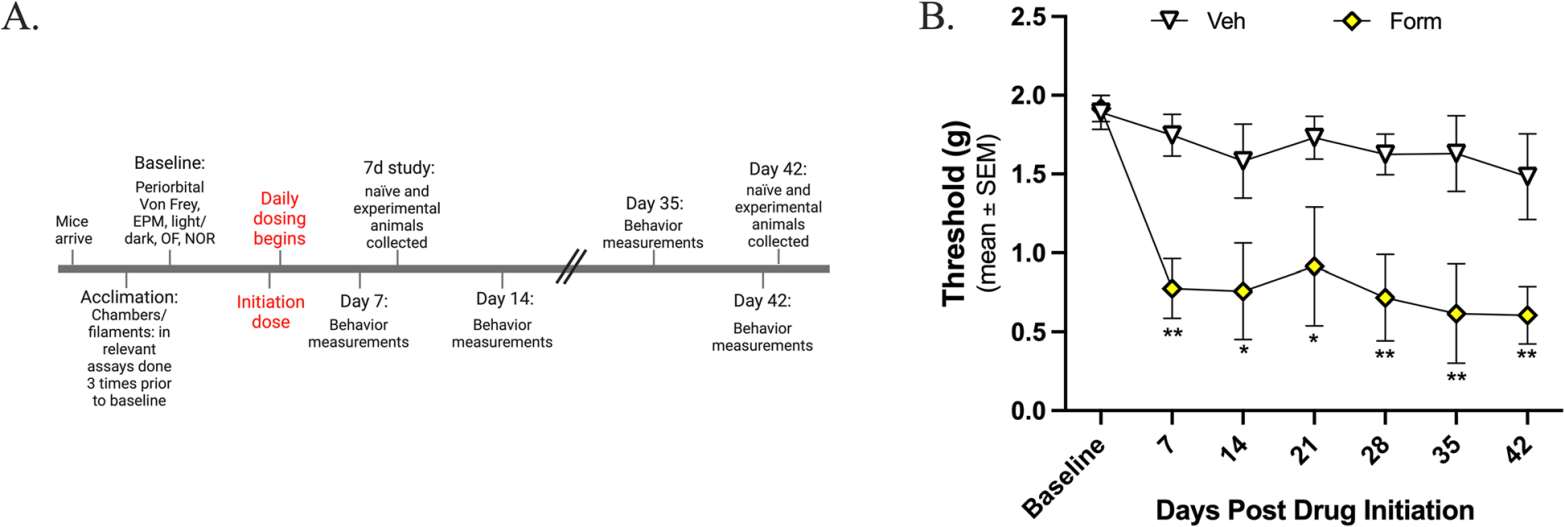
Formoterol induces periorbital allodynia 7 days after drug initiation out through 42 days of treatment. 8-week-old female C57Bl/6J mice were acclimated to the assay chambers and Von Frey filaments 3 times prior to baseline measurements; experimental measurements were taken weekly post-drug initiation for 42-days, formoterol (0.03 mg/kg, i.p.) or vehicle were administered daily for 42 days. **A** Timeline of experimental setting. **B** Formoterol treated mice began to exhibit headache-like periorbital allodynic behavior 7-days post drug initiation as compared to vehicle treated and maintained this periorbital sensitivity throughout the 42-day testing period. Data represented as mean of threshold (g) ±SEM. (*denotes *p*$\;\le\;$0.05, **denotes *p*$\;\le\;$0.01, compared to vehicle treated mice)

This experiment aimed to examine if formoterol induces headache-like periorbital allodynia behavior in mice and, if so, the degree to which this may occur. Formoterol (0.03 mg/kg, i.p.) or vehicle were administered daily for 7 or 42 days. Pre-drug baselines measurements were obtained prior to administration of the initiation dose of either formoterol or vehicle, after which experimental, post-drug values were measured weekly for up to 6 weeks. As hypothesized, there were no significant differences observed in the vehicle treated mice at any time point tested compared to baseline measurements (Fig. 1B; BL vs. vehicle, *p* > 0.05 at any time point, as assessed by two-way ANOVA with Tukey’s post-test, *n* = 6 in each group). However, in the formoterol treated mice there were persistent headache-like periorbital allodynic behaviors observed at almost all timepoints tested as soon as 7 days post drug administration (Fig. 1B; veh vs. form, 7 days: *p* = 0.0031, 14 days: *p* = 0.0113, 21 days: *p* = 0.0124, 28 days: *p* = 0.0055, 35 days: *p* = 0.0021, 42 days: *p* = 0.0072, as assessed by two-way ANOVA with Tukey’s post-test, *n* = 7 in each group; veh: mean ± SEM = 1.67 ± 0.04993, standard deviation = 0.1321; form: mean ± SEM = 0.8998 ± 0.1740, standard deviation = 0.4605). There were no significant differences in the formoterol treated mice between day 7 and day 42 (7-day vs 42-day: *p* = 0.5318, *n* = 6/timepoint), suggesting that while the functional outcome of the behavior does not change, the mechanism driving the behavior may be. These data suggest that formoterol does induce headache-like periorbital allodynic behaviors in the mice.

### Chronic administration of formoterol induces light sensitivity

**Fig. 2:**
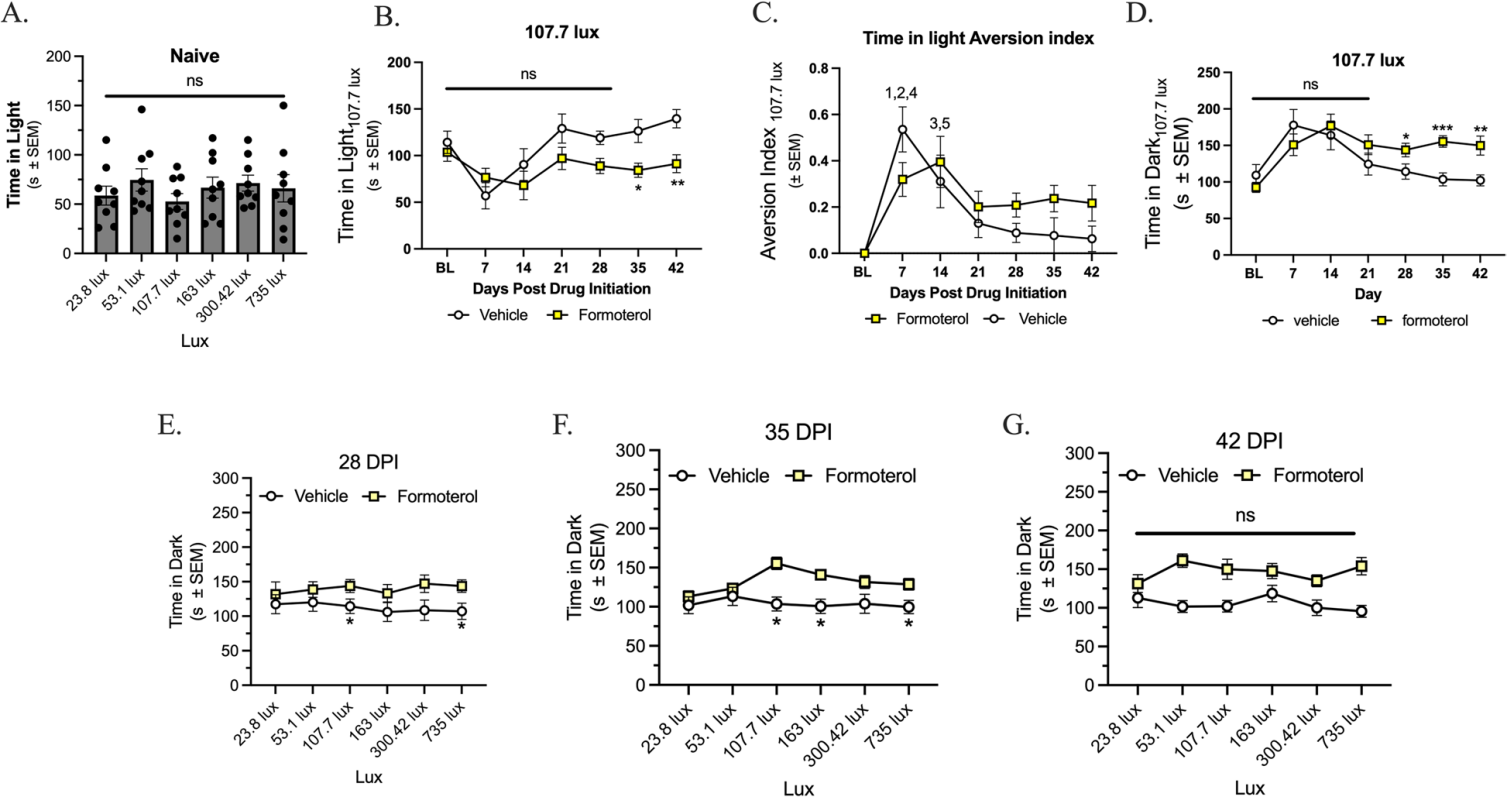
Chronic administration of formoterol induces photophobia as assessed via the light/dark box assay. 8-week-old female C57Bl/6J mice were acclimated to the assay chambers twice for 30m prior to baseline measurements, experimental measurements were then taken weekly post drug initiation for 42-days. Mice were tested at each lux (23.8, 53.1, 107.7, 163, 300.42, and 735) for 5m per lux. At all lux tested naïve mice showed no difference in time spent in light (**A**). At the 107.7 lux, formoterol treated mice spent less time in the light at days 35 and 42 (**B**), the light aversion index shows moderate habituation ay days 7 and 14 for both the formoterol and vehicle treated mice (**C**). Formoterol treated mice spent more time in the dark at days 28, 35 and 42 than the vehicle treated mice (**D**). In the lux curve, starting 28 days post drug initiation (DPI), formoterol treated mice spent significantly less time in the light at 23.8, 53.1, 107.7, 300.42 and 735 lux as compared to vehicle control (**E**). 35 DPI, formoterol treated mice spent significantly less time in light at lux 107.7 and 735 (**F**) and at 42 DPI, formoterol treated mice spent significantly less time in the light at lux 53.1, 107.7, 300.42 and 753 as compared to vehicle control (**G**). Chronic exposure to formoterol does induce light avoidance behaviors starting after 28 days of drug application. Data represented as time in light (s) ±SEM (*denotes *p*$\;\le\;$0.05, **denotes *p*$\;\le\;$0.01, ***denotes *p*$\;\le\;$0.001 compared to vehicle treated mice)

As discussed above, a frequently occurring comorbidity in headache patients is the presence of photophobia. In the first experiment, naïve female mice were exposed to 6 variable lux between 23.8 and 735 lux in a light/dark box assay, this was used as pre-drug baseline. No significant sensitivity to light between any of the tested lux was observed (Fig. 2A; *p* > 0.05, as assessed by one-way ANOVA with Tukey’s post hoc, *n* = 9; mean ± SEM = 65.02 ± 3.307, SD = 8.100). Mice were baselined at 107.7 lux. There was no difference in time in light at this lux until days 35 and 42 compared to their baseline (Fig. 2B; veh vs. form, 35 days: *p* = 0.0130, 42 days: *p* = 0.0045; veh: mean ± SEM = 110.8 ± 10.70, SD = 28.30; form: mean ± SEM = 87.09 ± 4.567. SD = 12.08). At days 7 and 14 post-drug initiation there was a decrease in light aversion in both vehicle and formoterol treated mice, this trend was not observed at any following timepoints (Fig. 2C; veh vs. form, (1) 7 days: *p* = 0.0333; BL vs. veh, (2) 7 days: *p* < 0.001, (3) 14 days: *p* = 0.0382; BL vs. form, (4) 7 days: *p* = 0.03, (5) 14 days: *p* = 0.0026; veh: mean ± SEM = 0.1720 ± 0.0709, SD = 0.187; form: mean ± SEM = 0.225 ± 0.0462, SD = 0.122). Continuing with 107.7 lux, formoterol treated mice showed an increase in time spend in the dark chamber on days 28, 35 and 42 post-drug initiation compared to vehicle treated mice (Fig. 2D, veh vs. form, 28 days: *p* = 0.0496, 35 days: *p* = 0.0004, 42 days: *p* = 0.0066; veh: mean ± SEM = 128 ± 11.5, SD = 30.5; form: mean ± SEM = 146 ± 9.70, SD = 25.7).

When comparing aversion at different lux, sensitivity was not observed until the 28-day timepoint, at which point formoterol treated mice spent less time in the 23.8, 53.1, 107.7, 300.42 and 735 lux (Fig. 2E; veh vs. form, 23.8 lux: *p* = 0.0436, 53.1 lux: *p* = 0.0478, 107.7 lux: *p* = 0.0464, 300.42 lux: *p* = 0.0237, 735 lux: *p* = 0.0241; veh: mean ± SEM = 93.00 ± 1.610, SD = 3.944; form: mean ± SEM = 123.7 ± 1.599, SD = 3.918). Sensitivity continued to be observed at the 35-day timepoint at 107.7 and 735 lux (Fig. 2F; veh vs. form, 107.7 lux: *p* = 0.0064, 735 lux: *p* = 0.0238; veh: mean ± SEM = 100.5 ± 3.769, SD = 9.233; form: mean ± SEM = 129.4 ± 2.109, SD = 5.165). Lastly, treatment with formoterol for 42-days showed a decrease in time spent in the light at the 53.1, 107.7, 300.42 and 753 lux (Fig. 2G; veh vs. form, 53.1 lux: *p* = 0.0017, 107.7 lux: *p* = 0.0005, 300.42 lux: *p* = 0.0069, 735 lux: *p* = 0.00002; veh: mean ± SEM = 94.56 ± 4.534, SD = 10.64, SEM = 4.342; form: mean ± SEM = 131.3, SD = 11.11, SEM = 4.534).

All data was assessed by two-way ANOVA with Tukey’s post-test, with an n of 10 in each group, data for 7-21DPI can be found in supplemental Figure 1.

### Formoterol does not induce anxiety-like behaviors

**Fig. 3:**
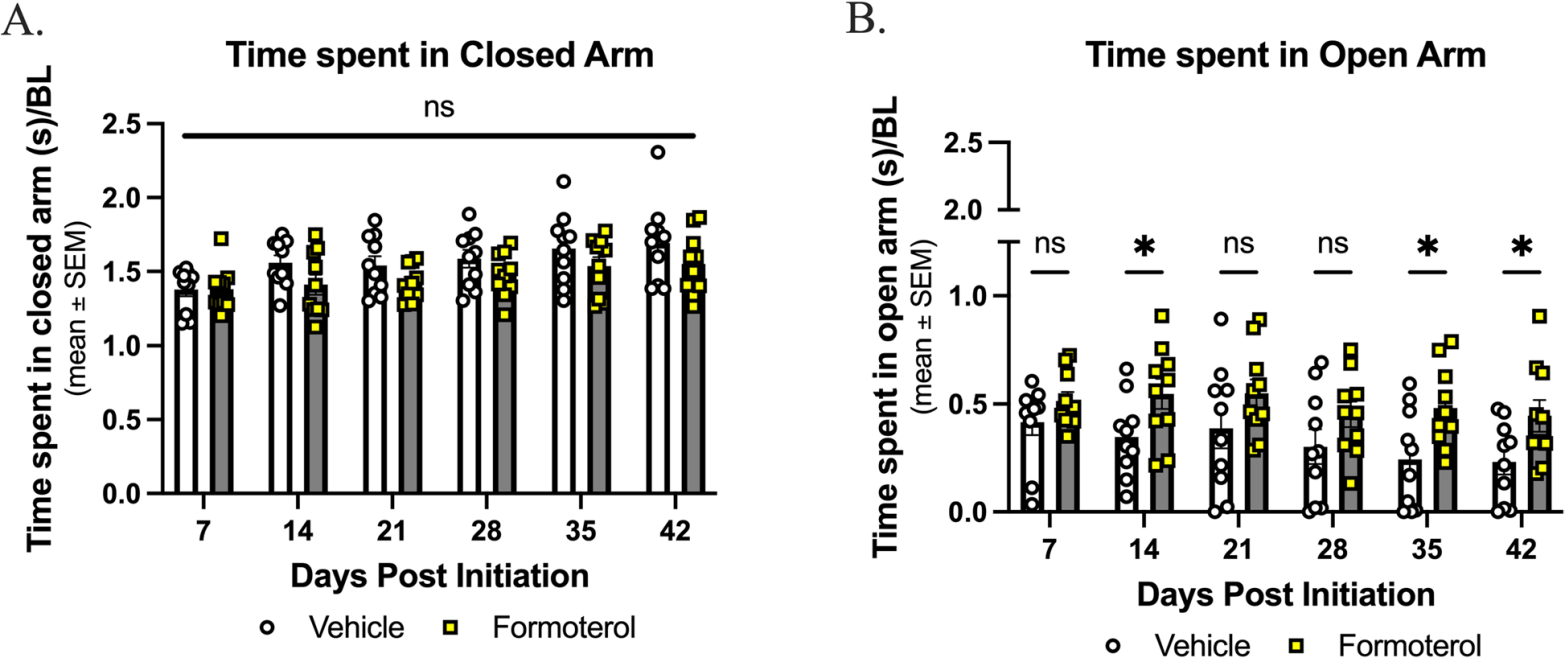
Anxiety-like behaviors are not observed under long-term treatment with formoterol. 8-week-old female C57Bl/6J mice were baselined for 300s/test prior to drug initiation; experimental measurements were taken weekly post-drug initiation for 42-days, formoterol (0.03 mg/kg, i.p.) or vehicle were administered daily for 42 days. **A** Throughout the 42-day study no difference between treatment groups was observed in terms of time spent in the closed arm. **B** Formoterol treated mice spent more time in the open arm at days 14, 35, and 42. Data represented as time spent in arm/baseline ± SEM. (*denotes *p*$\;\le\;$0.05, compared to vehicle control)

Anxiety is a common side-effect of formoterol as reported by the FDA, and a co-morbidity of chronic headache disorders [30, 58–60]. The elevated plus maze was used to assess anxiety like behavior with formoterol treatment. There was no difference observed between the formoterol-treated groups and the vehicle-treated groups in the closed arm (Fig. 3A; veh vs. form, *p* > 0.05 as assessed by two-way ANOVA with Tukey’s post-test, *n* = 10 in each group; veh: mean ± SEM = 1.569 ± 0.04470, SD = 0.1095; form: mean ± SEM = 1.464 ± 0.02862, SD = 0.07011). Formoterol treated mice spent more time in the open arm than vehicle treated mice at 14 days, 35 days and 42 days (Fig. 3B; veh vs. form, 14 days: *p* = 0.037, 35 days: *p* = 0.0131, 42 days: *p* = 0.0268 as assessed by two-way ANOVA with Tukey’s post-test, *n* = 10 in each group; veh: mean ± SEM = 0.3214 ± 0.03066, SD = 0.07509, SEM = 0.03066; form: mean ± SEM = 0.4986 ± 0.01874, SD = 0.04590). While individuals taking formoterol have reported experiencing anxiety, this is not reported in all patients. Despite these clinical observations, the presence of anxiety-like behavior was not observed in our animal model, the anxiety associated with formoterol treatment could remain patient-relevant.

### Age and formoterol treatment dynamically regulates levels of endocannabinoid lipids, 2-AG and AEA, and CB_1_R expression within the PAG in a time-dependent manner

**Fig. 4:**
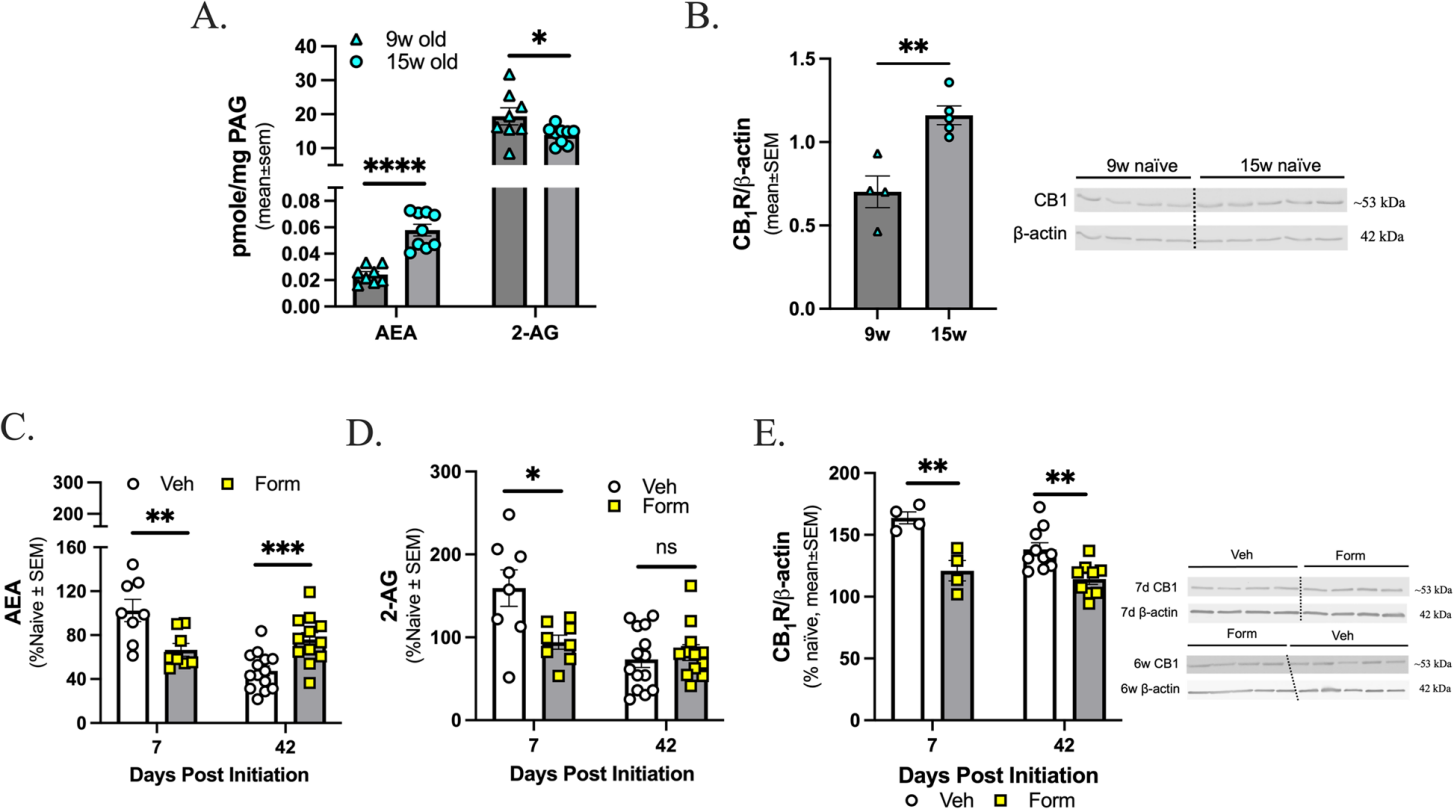
AEA, 2-AG, and CB_1_R expression in the PAG changes with age and with formoterol treatment. The PAGs from C57Bl/6J female age-matched naïve mice were harvested and eCB lipids assessed via LC-MS, (**A**) dynamic age-related changes were observed for both AEA and 2-AG. Data represented as mean ± SEM in pmol/mg unit. **B** In the naïve mice, protein expression of cannabinoid receptor 1 (R) changes with age. Data represented as mean ± SEM. β-actin was used as loading control. **C** 7d post drug initiation, AEA levels are significantly decreased in formoterol treated mice versus vehicle treated mice but at 6w post drug initiation AEA levels are significantly increased in formoterol treated mice. **D** 2-AG level within the PAG is significantly decreased in formoterol treated mice versus vehicle treated mice at 7-days, but by 42-days there is no difference observed between experimental groups. **E** expression within PAG in formoterol treated mice is significantly decreased as compared to vehicle treated mice both 7d and 6w post drug initiation. Data represented as percentage of naïve ± SEM. (*denotes *p*$\;\le\;$0.05, **denotes *p*$\;\le\;$0.01, ***denotes *p*$\;\le\;$0.001, **** denotes *p*$\;\le\;$0.0001 compared to age-matched or vehicle treated mice)

Previous work suggests that levels of 2-AG are reduced within the PAG, but not the TG or Vc, during cortical spreading depression-associated allodynia and MOH models using sumatriptan and morphine [25, 26]. Given the duration of the study, the first experiment investigated potential age-related differences within PAG with respect eCB levels and CB_1_ receptor expression in naïve mice; the following ages were assessed: 9-weeks-old, to model the 7-day time point, and 15-weeks-old, to model the 42-day duration. AEA lipid levels were higher in the 15-week-old mice as compared to the 9-week-old mice. In contrast, 2-AG lipid levels were lower in the 15-week-old mice as compared to the 9-week-old mice (Fig. 4A; 9-week vs 15-week, AEA: *p* < 0.0001, 2-AG: *p* = 0.0479, as assessed by unpaired t-test, *n* = 8–9; AEA: 9w: mean ± SEM = 0.02424, SD = 0.006535, SEM = 0.002311; 15w: mean ± SEM = 0.05787, SD = 0.01334, SEM = 0.004445; 2-AG: 9w: mean ± SEM = 19.37, SD = 7.149, SEM = 2.527; 15w: mean ± SEM = 13.90, SD = 2.549, SEM = 0.8495). Additionally, protein expression of CB_1_R was significantly higher in the 15-week-old mice versus the 9-week-old mice (Fig. 4B; 9-week vs 15-week, *p* = 0.0033 as assessed by unpaired t-test, *n* = 4–5; 9w: mean ± SEM = 0.7019, SD = 0.1906, SEM = 0.09530; 15w: mean ± SEM = 1.161, SD = 0.1253, SEM = 0.05605).

These results indicate that rigorous assessment of eCB levels and CB_1_R protein expression in formoterol or vehicle treated mice required results be normalized to their specific age-matched naive controls. Mice were treated for either 7- or 42-days with formoterol or vehicle starting at 8-weeks of age. After 7-days of formoterol treatment, there was a significant decrease in levels of AEA within the PAG versus the vehicle treated mice; after 42-days of formoterol treatment, there was a significant increase in levels of AEA versus the vehicle treated mice (Fig. 4C; veh vs. form, 7 days: *p* = 0.0083 *n* = 8, 42 days: *p* = 0.0010 *n* = 12–14 as assessed by unpaired t-test; 7d AEA: veh: mean ± SEM = 102.4, SD = 28.60, SEM = 10.11; form: mean ± SEM = 66.57, SD = 16.54, SEM = 5.847; 42d AEA: veh: mean ± SEM = 47.45, SD = 16.92, SEM = 4.523; 42d: mean ± SEM = 76.04, SD = 21.87, SEM = 6.313). Regarding 2-AG, after 7-days of formoterol mice exhibited a decrease in levels of 2-AG within the PAG as compared to vehicle control; however, this reduction of 2-AG level was not observed at the 42-day timepoint (Fig. 4D; veh vs. form, 7 days: *p* = 0.0151 *n* = 8, 42 days: *p* > 0.05 as assessed by unpaired t-test; 7d 2-AG: veh: mean ± SEM = 159.3, SD = 62.17, SEM = 21.98; form: mean ± SEM = 94.11, SD = 23.94, SEM = 8.463; 2-AG: veh: mean ± SEM = 73.30, SD = 36.10, SEM = 9.649; form: mean = 81.62, SD = 32.74, SEM = 9.451). At both the 7-day and 42-day timepoints there was a decrease in CB_1_R protein expression in the formoterol treated mice versus the vehicle treated mice (Fig. 4E; veh vs. form, 7 days: *p* = 0.0042 *n* = 4 per group, 42 days: *p* = 0.0033 *n* = 9–10 per group as assessed by unpaired t-test; 7d: veh: mean ± SEM = 163.7, SD = 9.603, SEM = 4.802; form: mean ± SEM = 120.9, SD = 16.52, SEM = 8.261; 42d: veh: mean ± SEM = 138.2, SD = 16.91, SEM = 5.346; form: mean ± SEM = 114.3, SD = 13.24, SEM = 4.414). Whole blot representations are in Supplemental Figures 2 and 3, raw densitometry values can be found in Supplemental Figure 4. All together, these results suggest that treatment with formoterol dynamically regulates eCB tone within the PAG.

### ADRB2 receptor expression does not change with age but is altered by chronic treatment with formoterol

**Fig. 5:**
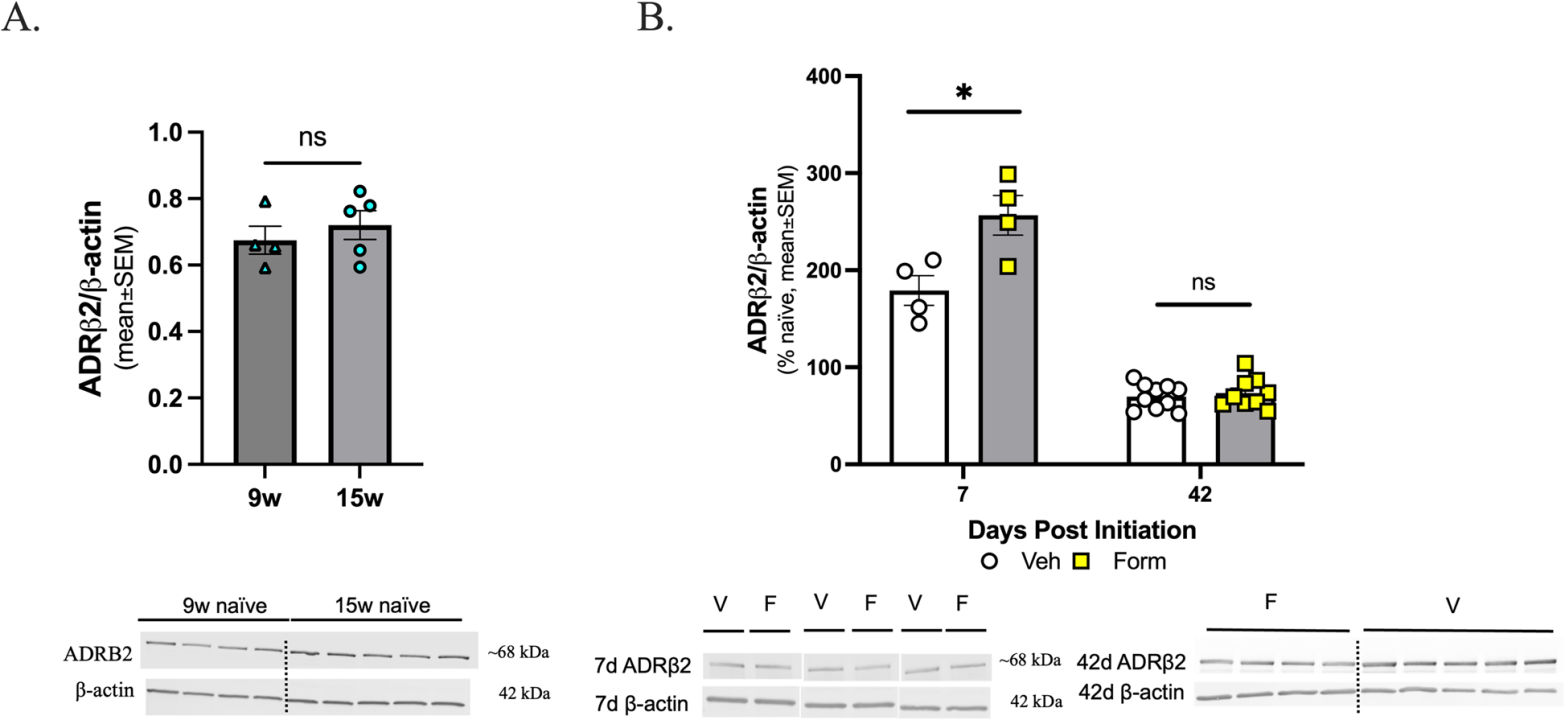
Formoterol treatment induced changes in the expression of ADRB2 receptor within the PAG 7-days post-initiation. The PAGs from C57Bl/6J female age-matched naïve mice were harvested and ADRB2 receptor protein expression assessed by Western immunoblotting, (**A**) age-related changes were not observed. Data represented as mean ± SEM. β-actin was used as loading control. **B** At 7 DPI, there is a significant increase in the expression of ADRB2 receptor in the formoterol treated mice versus the vehicle treated mice. No significant difference was observed in the expression of ADRB2 receptor 42 DPI, compared to vehicle control. Data represented as percentage of naïve ± SEM. β-actin was used as loading control. (*denotes *p*$\;\le\;$0.05 compared to age-matched or vehicle treated mice)

The protein expression of the ADRB2 within the PAG region, the target receptor of formoterol was assessed by Western immunoblotting. The ADRB2 expression was not dynamically regulated with age in PAG tissue (Fig. 5A; 9-week vs 15-week, *p* > 0.05, as assessed by unpaired t-test, *n* = 4–5; 9w: mean ± SEM = 0.6749, SD = 0.08414, SEM = 0.04207; 15w: mean ± SEM = 0.7202, SD = 0.09640, SEM = 0.04311). There was a significant increase in protein expression at the 7-day timepoint, which was diminished by the 42-day timepoint (Fig. 5B; veh vs. form, 7 days: *p* = 0.0227, 42 days: *p* > 0.05, as assessed by unpaired t-test, 7 days *n* = 4 per group, 42 days *n* = 9–10; 7d: veh: mean ± SEM = 175.2, SD = 34.65, SEM = 17.32; form: mean ± SEM = 256.6, SD = 40.60, SEM = 20.30; 42d: veh: mean ± SEM = 69.91, SD = 12.90, SEM = 4.080; form: mean ± SEM = 73.49, SD = 15.37, SEM = 5.123). This effect was not unexpected, as repeated agonism of a G protein-coupled receptor can initially cause an increase in receptor expression, followed by a decrease due to desensitization of the receptor [61]. Whole blot representations are in Supplemental Figures 2 and 3, raw densitometry values can be found in Supplemental Figure 4.

### Targeting of the endocannabinoid system reverses the observed formoterol-induced periorbital allodynia and alters levels of AEA

**Fig. 6:**
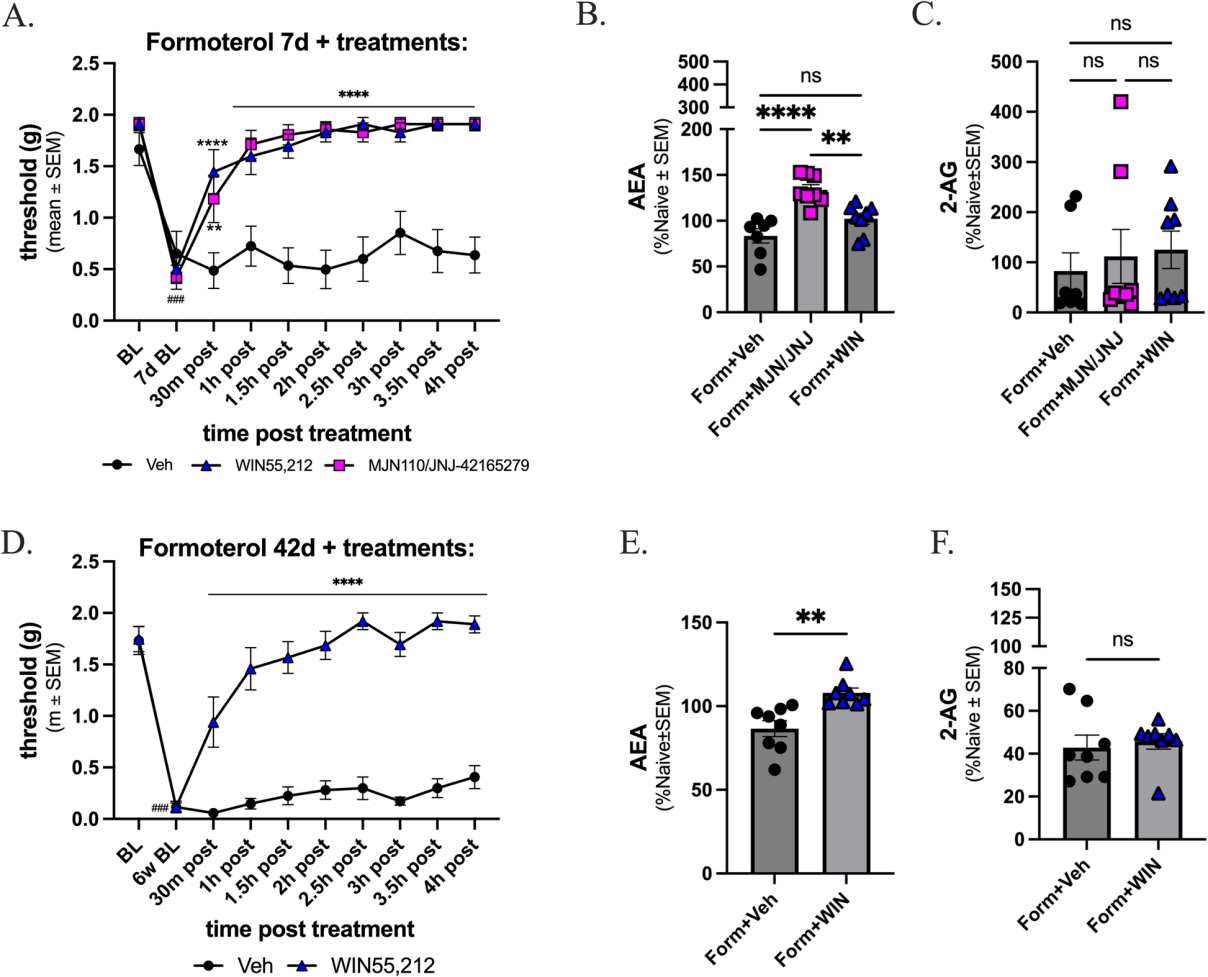
MAGL/FAAH and CB_1_R inhibitors reversed formoterol induced periorbital allodynia. 8-week-old female C57Bl/6J mice were acclimated to the assay chambers and Von Frey filaments 3 times prior to baseline measurements, followed by treatment with formoterol for 7 days or 42 days. Mice given formoterol for 7 days received either MJN/JNJ, WIN,55,212-2, or vehicle on the last day of formoterol administration. **A** At time of baseline, mice did not exhibit headache-like periorbital allodynic behaviors; after receiving formoterol for 7 days, increase in periorbital sensitivity was observed. **A** 30-minutes after administration of MJN/JNJ or WIN, a significant decrease in headache-like periorbital allodynic behavior was observed, with no significant difference in threshold values between day 0 baseline and 1 hour post inhibitor administration for these treatments. **B** MJN/JNJ administration induced significant increase in the level of AEA within the PAG compared to vehicle control. **C** There are no significant changes observed in the levels of 2-AG in either treatment group compared to vehicle control. A second cohort of mice received formoterol for 6 weeks and were then given either WIN or vehicle. PAG was harvested 5 hours later. **D** In the 6w study, 30 minutes after administration of WIN 55,212, a significant decrease in headache-like periorbital allodynic behaviors was observed but values did not reach pre-drug administration baseline values until 2.5 hours post-treatment with WIN55,212. In a separate set, mice received formoterol daily for 7 days, at which point they received either MJN/JNJ, WIN, or vehicle. PAG was harvested after 5 hours and subjected to LC-MS to measure endocannabinoid levels. **E** WIN treated mice exhibited an increase in levels of AEA versus vehicle treated mice, and (**F**) levels of 2-AG were not significantly different between the two groups. Data represented as threshold (g) ± SEM or percentage of naive ± SEM. (### denotes *p*$\;\le\;$0.001 compared to Day 0 BL; **denotes *p*$\;\le\;$0.01, **** denotes *p*$\;\le\;$0. compared to form+veh)

Given that endocannabinoid lipid tone (2-AG and AEA) and CB_1_R expression were reduced on Day 7 and that AEA was increased over controls on Day 42 of formoterol-induced headache (Fig. 4), the next study asked if normalizing eCB tone using pharmacological inhibitors of 2-AG degradation, MAGL (MJN) and AEA degradation, FAAH (JNJ) or a non-selective CB_1_/CB_2_R agonist (WIN55,212-2), could mitigate the behaviors at these time points after a single bolus. All mice received formoterol daily for 7- or 42-days with periorbital allodynia behavior assessed prior to drug administration. On the final day of formoterol administration (D67 or D42), periorbital allodynia was reassessed prior to inhibitor/agonist injection and every 30-minutes post injection (PI) for 4-hours. Prior to drug administration, all three groups showed periorbital sensitivity compared to the baseline measurements (Fig. 6A; baseline vs. all treated groups, PI 7d BL: *p* < 0.001; Veh BL: mean ± SEM = 1.666, SD = 0.4219, SEM = 0.1594, Veh 7d BL: mean ± SEM = 0.6514, SD = 0.5737, SEM = 0.2168, MJN/JNJ BL: mean ± SEM = 1.962, SD = 0.09390, SEM = 0.03833, MJN/JNJ 7d, BL: mean ± SEM = 0.4517, SD = 0.3301, SEM = 0.1348; WIN BL: mean ± SEM = 1.918, SD = 0.2009, SEM = 0.08200, WIN 7d BL: mean ± SEM = 0.6600, SD = 0.3327, SEM = 0.1358). 30-minutes after drug application, reversal of formoterol-induced periorbital allodynia was observed in both treatment groups, compared to vehicle control (form + veh vs. form + MJN/JNJ, 30-minutes PI: *p* = 0.0020, MJN/JNJ 7d BL: mean ± SEM = 0.4517, SD = 0.3301, SEM = 0.1348, MJN/JNJ 30m: mean ± SEM = 1.088, SD = 0.6590, SEM = 0.2691; WIN 7d BL: mean ± SEM = 0.6600, SD = 0.3327, SEM = 0.1358, WIN 30m: mean ± SEM = 1.088, SD = 0.6590, SEM = 0.2691; 1-hours through 4-hours PI: *p* < 0.0001; form + veh vs. form + WIN, 30-minutes through 4-hours PI: *p* < 0.0001; veh: mean ± SEM = 0.7324, SD = 0.3463, SEM = 0.1095; MJN/JNJ: mean ± SEM = 1.645, SD = 0.4830, SEM = 0.1527; WIN: mean ± SEM = 1.654, SD = 0.4340, SEM = 0.1372). All data was assessed by two-way ANOVA with Tukey’s post-test, with an n OF 8 in each group.

Levels of AEA and 2-AG within the PAG were then assessed at both timepoints described above. At the 7-day timepoint, there was a significant increase in levels of AEA in the MJN/JNJ treated mice compared to vehicle control (Fig. 6B; form + veh vs. form + MJN/JNJ, *p* < 0.0001, as assessed by one-way ANOVA with Tukey’s posttest, *n* = 7–8 per group; veh: mean ± SEM = 83.51, SD = 20.63, SEM = 7.796; MJN/JNJ: mean ± SEM = 133.8, SD = 15.88, SEM = 5.614). No significant changes in 2-AG level were detected in the MJN/JNJ treated group compared to vehicle control (Fig. 6C; form + veh vs. form + MJN/JNJ, *p* > 0.05, as assessed by one-way ANOVA with Tukey’s posttest, *n* = 7–8 per group; veh: mean ± SEM = 82.62, SD = 96.04, SEM = 36.30; MJN/JNJ: mean ± SEM = 111.8, SD = 152.3, SEM = 53.84;). The WIN55,212 treatment did not induce significant changes in either endocannabinoid level at day 7 compared to vehicle control (Fig. 6B and **C**; form + veh vs. form + WIN, 7d PI: *p* > 0.05 as assessed by one-way ANOVA, *n* = 7-8per group; AEA: mean ± SEM = 102.2, SD = 16.64, SEM = 5.882; 2-AG: mean ± SEM = 125.1, SD = 105.4, SEM = 37.27).

At the 42-day timepoint when CB_1_R expression remained downregulated, a separate cohort of mice were given vehicle or WIN55,212 and behavior was assessed as above. Only WIN55,212 was administered at this time based on the LC-MS and WB data, showing an increase in the levels of AEA and no difference in the levels of 2-AG in the formoterol treated mice, suggesting the degradation enzymes MAGL and FAAH are not playing major roles in headache-like periorbital allodynic behavior at this later timepoint (Fig. 4). At day 42, mice exhibited periorbital allodynia as compared to pre-drug baseline (Fig. 6D; baseline vs. all treated groups, PI 42d BL: *p* < 0.0001; BL: mean ± SEM = 1.733, SD = 0.3840, SEM = 0.1358, form + Veh 7d BL: mean ± SEM = 0.1163, SD = 0.1602, SEM = 0.05663, BL: mean ± SEM = 1.747, SD = 0.3501, SEM = 0.1238, form + WIN 42d BL: mean = 0.1163, SD = 0.1113, SEM = 0.03937). Vehicle administration did not alter facial withdrawal thresholds at any time point evaluated. In contrast, 30 minutes post administration of WIN55,212, periorbital allodynia was significantly reversed, and this maintained throughout the four hour test period (Fig. 6D; form + veh vs. form + WIN, 30-minutes through 4-hours PI: *p* < 0.0001; veh: mean ± SEM = 0.3735, SD = 0.4886, SEM = 0.1545, WIN: mean ± SEM = 1.494, SD = 0.5646, SEM = 0.1785). All data was assessed by two-way ANOVA with Tukey’s post-test, with an n of 8 in each group.

At the 42-day timepoint there was an increase in levels of AEA observed in the WIN treated mice versus the vehicle treated mice (Fig. 7E; form + veh vs. form + WIN, 42d PI: *p* = 0.0018 as assessed by unpaired t-test, *n* = 8 per group; veh: mean ± SEM = 86.56, SD = 13.56, SEM = 4.796, WIN: mean ± SEM = 107.9, SD = 8.027, SEM = 2.838). There was also no difference in levels of 2-AG in the WIN treated mice versus vehicle control (Fig. 6F; form + veh vs. form + WIN, 42d PI: *p* > 0.05 as assessed by unpaired t-test, *n* = 8 per group; veh: mean ± SEM = 42.87, SD = 16.37, SEM = 5.787, WIN: mean ± SEM = 45.77, SD = 10.23, SEM = 3.618). These data suggest that the endocannabinoid system plays a role in formoterol-induced headache-like periorbital allodynic behaviors, supporting the hypotheses that there is an interaction occurring between the endocannabinoid and adrenergic system within PAG.

## Discussion

One reported side effect of formoterol, an FDA-approved drug known to cross the BBB [31, 32], is headache. With current efforts to repurpose formoterol for various disorders, addressing this adverse effect could prove clinically important [37, 39, 40, 62–64]. The studies above revealed that daily formoterol administration induced periorbital allodynia within 7-days post drug initiation, and that this periorbital allodynic behavior was maintained across 42-days. Additionally, animals exhibited photosensitivity after chronic treatment with the drug. Though anxiety is a known side-effect of formoterol and is a common co-morbidity of chronic headache [30, 58–60], anxiety-like behaviors were not observed in the animals receiving formoterol. The behavioral results confirmed the presence of headache related behaviors in the formoterol treated mice, however the mechanism behind this behavior has not been reported. Herein, the mechanism behind formoterol-induced periorbital allodynic behaviors is hypothesized to involve the endocannabinoid system. Age and formoterol were shown to dynamically regulate endocannabinoid levels and CB_1_R expression in the PAG, suggesting there is eCB system involvement in the observed headache-like periorbital allodynic behaviors. When different aspects of the eCB system were targeted in formoterol treated mice, a reversal of the induced periorbital allodynia was observed. These results further support the hypothesis that formoterol is interacting with, or acting through, the endocannabinoid system to induce these behaviors.

Headache has high prevalence in females and is reported as greatly interfering with quality of life [1, 4]. One of the currently proposed mechanisms for headache and migraine include irregularities in neuromodulator release and uptake, including those within the eCB system [13]. As such, investigating this system within the context of formoterol-induced headache could provide insight for treatment and prevention options for patients currently using the drug. In a general sense, the characterized use of formoterol is as a long-lasting selective β_2_-adrenergic receptor (ADRB2) agonist acting as a bronchodilator for use in cases of obstructive pulmonary disease and severe asthma [43]. As it is FDA-approved, a record of known off-target effects are already reported. These include interference with cardiac function resulting in increased heart rate, QT prolongation, and reduction in plasma potassium levels in a subset of patients [65] and side-effects such as anxiety and headache. Additionally, migraine and asthma are considered comorbid chronic disorders that can be challenging to manage pharmacologically due to their episodic natures and their unresponsiveness to the currently available treatment options [66, 67].

Research has reported that many abortive pain medications are capable of inducing medication overuse headache (MOH), including but not limited to several drug classes such as analgesics (e.g. opioids), triptans, and ergots [68–70]. The probability of experiencing an MOH is increased in patients taking triptans or narcotics, even if the medication overuse was only for a short period [69–71]. Common comorbidities that occur with headache include photosensitivity, anxiety, mobility issues and learning deficits [60, 72–77]. In general, a link between the sensitization of adrenergic receptors and an increase in anxiety-like behaviors and panic attacks has been reported in patients; of note, the FDA lists anxiety as a possible side-effect of formoterol [30, 58]. Chronic formoterol treatment induced the headache-like periorbital allodynic behaviors, periorbital allodynia and light sensitivity in female mice, with minor impacts on motor movements, and no impact on anxiety-like behaviors and cognition. Other models of MOH using sumatriptan and morphine have shown similar timelines as tested here in the induction of headache-like periorbital allodynic behaviors [13, 78]. Other models of MOH, such as a model using morphine, have shown that light sensitivity commonly accompanies MOH [71, 79]. Rare cases of photosensitivity have been reported in some cases of treatment with α_2_-adrenergic receptor agonists rilmenidine and methyldopa, but this has yet to be reported with formoterol treatment [80]. Additionally, a study using cane toads investigated the inducibility of photosensitivity by adrenergic receptor agonists and found that treatment with the β-adrenergic agonist isoprenaline induced the most pupil dilation [81]. Considering there is an increased risk of photocarcinogenic effects that can accompany photosensitivity when followed with ultraviolet or visible light, new insight into the effects of formoterol treatment on photosensitivity could provide new treatment considerations [82]. Lastly, while studies have reported a relationship between anxiety levels and β-adrenergic receptor function in human patients where sensitization of ADRBs in ‘normal’ patients correlated to an increase in anxiety-like behavior [58], this behavior was not observed in the mice assessed via elevated plus maze herein. Learning and mobility were also assessed via the following assays in formoterol treated mice: novel object recognition, open field, and Rotorod. Treatment with formoterol did not induce learning deficits at any timepoint tested (Supplemental Figure 4A), and while decreases in distance travelled were observed (Supplemental Figure 4B) there were no changes in mobility when compared to naïve mice (Supplemental Figure 4C). This suggests that formoterol may be inducing a headache-like outcome that is not coupled with other comorbidities.

The endocannabinoid system has been shown to be involved in homeostasis [83], and age-related changes in receptor expression CB_1_ and ADRB2 have been reported in other CNS models [84]. The data presented above support these observations and report age-dependent expression of CB_1_R within the PAG of adult female C57Bl/6J mice. Of important note, dysregulation of the endocannabinoid system has been implicated in the development and progression of chronic migraine and MOH, both preclinically and clinically [13, 85]. Specifically, low levels of AEA and 2-AG have been shown to play roles in the development and persistence of headache in rodent models [13, 25, 26]. The mechanism hypothesized to be at play here in formoterol-induced headache is the involvement of endocannabinoid system in the PAG. Endocannabinoid lipid levels in the PAG have been shown to be altered in cases of MOH [25]. Evaluating levels of endocannabinoid lipids AEA and 2-AG within the PAG, LC-MS analysis showed that levels of both eCB lipids were dynamically regulated by age and with formoterol. To further investigate the possible involvement of the eCB in the formoterol-induced headache mechanism, the eCB system was targeted using inhibitors of the eCB enzymes FAAH and MAGL, as well as an eCB receptor targeting agent. The reversal of the headache-like periorbital allodynic behaviors observed herein is a phenomenon that has also been observed previously in models of migraine using different pharmacologic agents

While current data suggest a relationship between ADRB2 and the eCB system, this study does not differentiate between the effects of the FAAH and MAGL inhibitors, nor the receptors. Further work would aim to parse out these differences. For example, formoterol-induced headache could be due to opposing mechanisms of the endocannabinoid and adrenergic systems as CB_1_R has been linked to G_i/o_ activation, whereas formoterol canonically acts on G_s_-proteins [86, 87]. Alternatively off target effects may be implicated such as WIN 55,212 that act on the CB receptors have been reported to affect neuronal Na^+^,K^+^-ATPase via activation of these proteins [88]. As formoterol is known to exert action on vascular cells throughout the body [89, 90] it is more than likely that formoterol is also acting on vascular cells within the CNS. As it is a vasodilator, and a subtype of immediate headache have been connected to nitric oxide mediated vasodilation, this may be the mechanism initially at play [91]. However, our hypothesized interaction between the eCB and β2-adrenergic receptor could help explain the sustained periorbital allodynic behavior observed. Additionally, formoterol has been reported to interact with chemokines and cytokines [35], glial cells [32], and neurons [31, 92]. However, the mechanism(s) by which this occurs has not been elucidated. Based on these works and the presence of CB_1_ receptors on endothelial and smooth muscle cells in cerebral vessels [93], it is possible that the site of action for the observed outcomes is mediated by vascular cells. Additionally, for the periorbital von Frey an ambient light lux of 354 was used; at this lux the separate light/dark study cohort showed sensitization at day 28 post-drug initiation; future work will utilize a lower lux that does not show sensitization at any timepoint. Lastly, female mice were the focus of this study as medication overuse headache and asthma have been reported to occur more frequently within the female human population [94, 95] and no sex differences in long lasting beta agonists have been identified [96]. While these possible alternative interpretations do not negate the data presented herein, it does provide a direction for future work aiming to determine the molecular and cellular mechanisms underlying formoterol-induced headache-like periorbital allodynic behavior including potential sexual dimorphism.

## Conclusions

The study presented herein shows that daily intraperitoneal treatment with formoterol induces periorbital allodynia, which can be reversed by targeting MAGL, FAAH, and agonism of eCB receptors, and decreases levels of endogenous cannabinoids AEA and 2AG at 7-days post drug initiation and increases levels of AEA at the 42-day timepoint. This study focused on evaluating a single brain region known to play a role in the pathophysiology of headache, future work aims to assess possible changes in other brain regions of interest including: the fifth cranial nerve, the trigeminal nerves, and the visual cortex, all of which are important brain regions in pain processing [13]. As AEA is also a vanilloid receptor agonist [97], future studies will include investigation into non-CBR mechanisms while still addressing alterations in eCB tone. MJN 110 also acts to inhibit alpha/beta-hydrolase domain containing 6 (ABHD6), which our research group has shown plays a role in cortical spreading depression induced periorbital allodynia in rats [26]. As such, this is an additional avenue of investigation we aim to take in future work. Additionally, sex differences have been shown in literature to contribute to difference in prevalence of headache in different sex populations [98], as well as altered pharmacological responses [99]. In future, this aspect will be investigated in respect to formoterol-induced headache by assessing the pain response in males upon treatment with formoterol and both timepoints assessed. Formoterol has been shown to alter chemokine and cytokine expression [35]. In a study using human serum samples, levels of proinflammatory cytokines were increased in those patients experiencing active migraine attacks versus the healthy controls [100]. This would be another facet to investigate both within the PAG, but also within the other brain regions of interest. Current observations suggest that the endocannabinoid system is playing a role in formoterol associated side-effects, such as headache; given the current push in literature to repurpose the drug for other disorders, investigation into dual targeting systems should be pursued.

## Supplementary Information

Supplementary Material 1.

Supplementary Material 2.

Supplementary Material 3.

Supplementary Material 4.

Supplementary Material 5.

## Data Availability

The datasets used and/or analyzed during the current study are available from the corresponding author on reasonable request.

## Abbreviations

2-AG: 2-arachidonoylglycerol
ADRB2: β_2_-adrenergic receptor
AEA: Anandamide
BBB: Blood brain barrier
BL: Baseline
BSA: Bovine serum albumin
CB_1_/_2_R: Cannabinoid receptor1/2
DMSO: Dimethyl sulfoxide
DPI: Days post drug initiation
eCB: Endocannabinoid system
FAAH: Fatty acid amide hydrolase
Formoterol/form: Formoterol fumurate dihydrate
JNJ: JNJ-42165279
LC-MS: Liquid chromatography mass spectrometry
MAGL: Monoacylglycerol lipase
MJN: MJN110
MOH: Medication overuse headache
PAG: Periaqueductal grey
PI: Post-injection
SCI: Spinal cord injury
TBS: Tris-buffered saline solution
TBST: Tris-buffered saline solution + 1% Tween20
Veh: Vehicle
WB: Western blot
WIN: WIN 55,212
