# Supplementary figures and images for: Formoterol dynamically alters endocannabinoid tone in the periaqueductal gray inducing headache
Source: J Headache Pain. 2024 Nov 19;25(1):200. doi: 10.1186/s10194-024-01907-y (PMC11575070; doi:10.1186/s10194-024-01907-y)

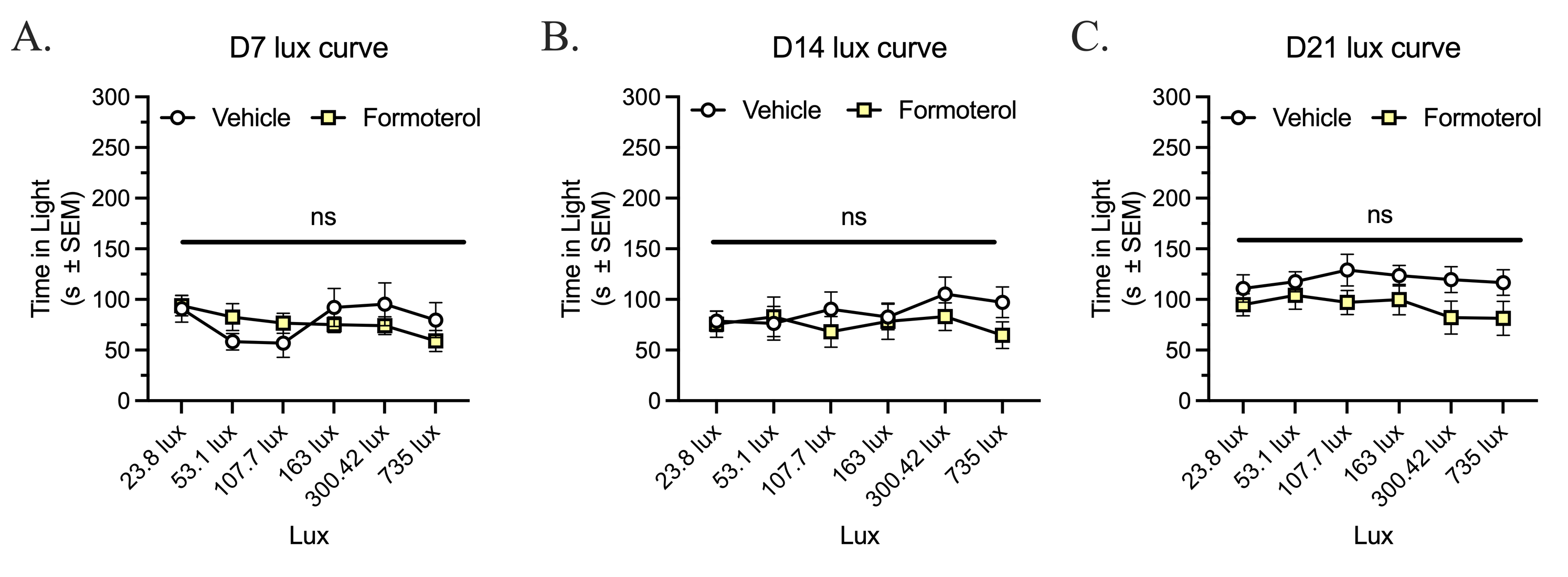

Supplement: Supplementary file 1 — Supplementary Material 1. [file 10194_2024_1907_MOESM1_ESM.tiff]

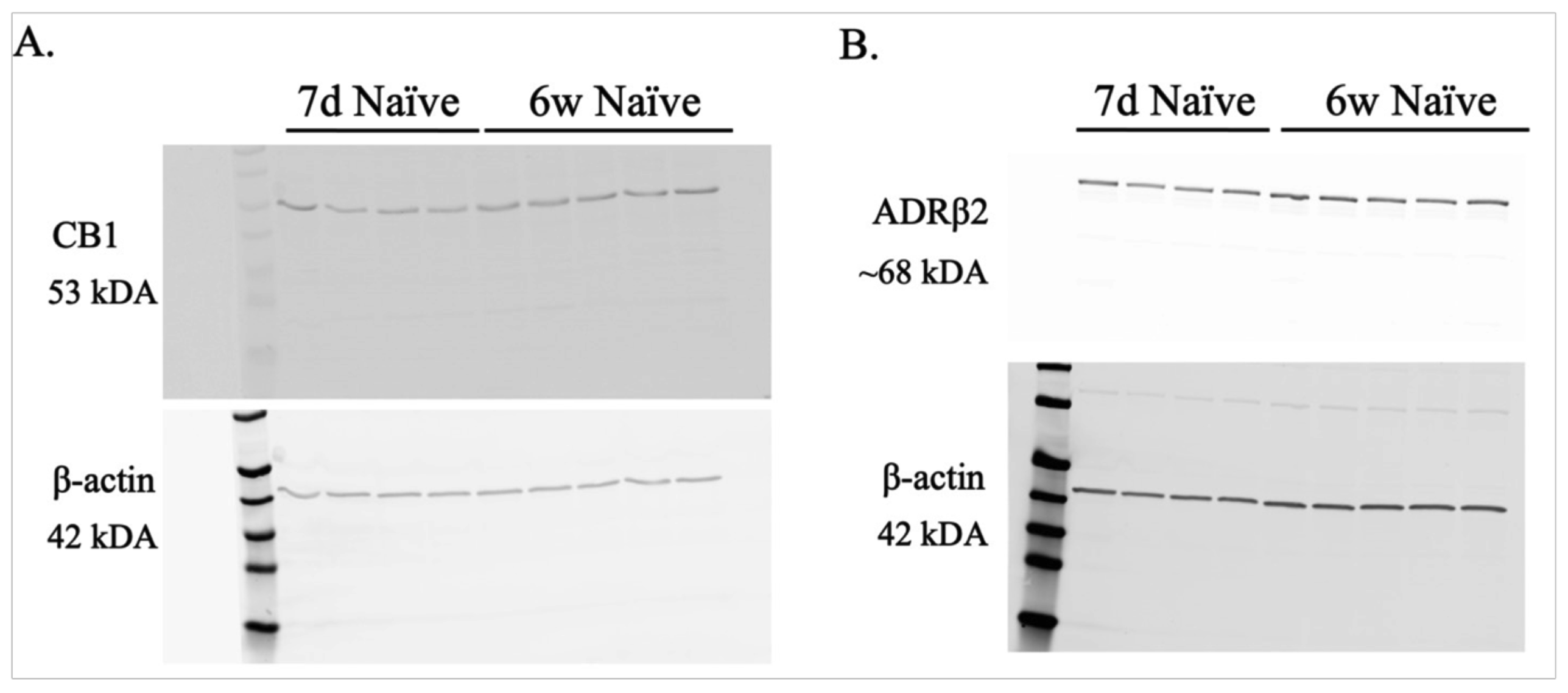

Supplement: Supplementary file 2 — Supplementary Material 2. [file 10194_2024_1907_MOESM2_ESM.tiff]

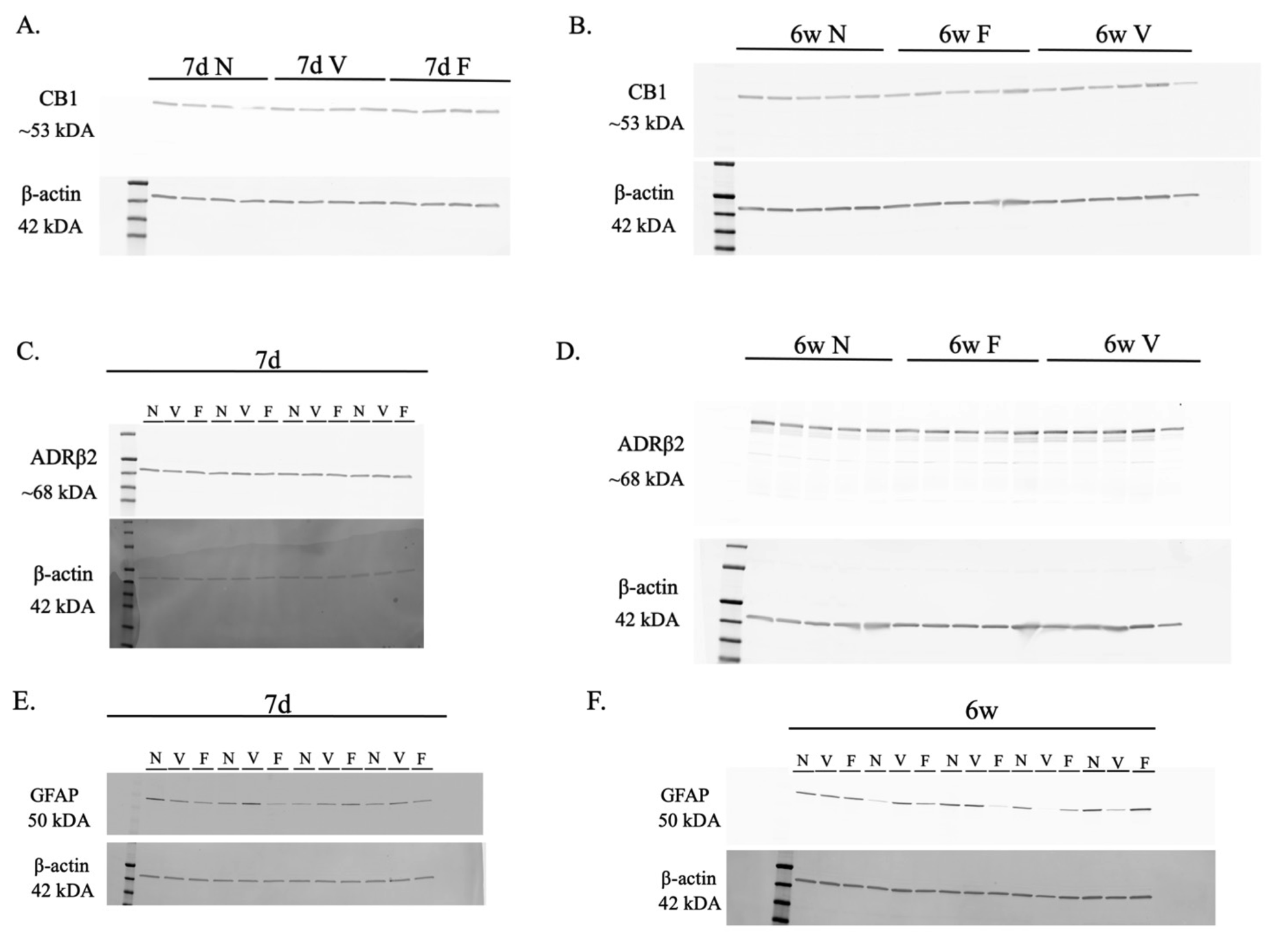

Supplement: Supplementary file 3 — Supplementary Material 3. [file 10194_2024_1907_MOESM3_ESM.tiff]

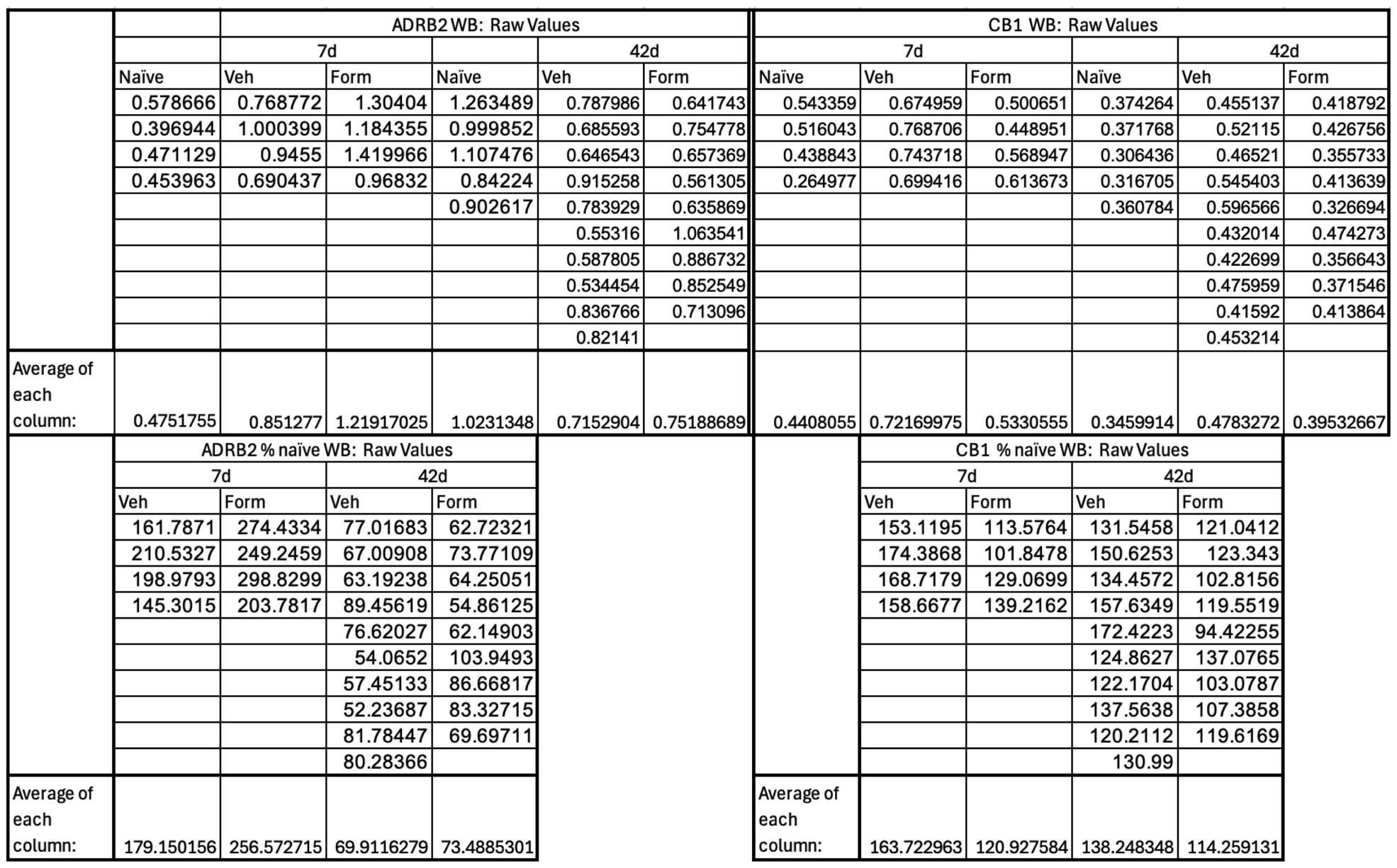

Supplement: Supplementary file 4 — Supplementary Material 4. [file 10194_2024_1907_MOESM4_ESM.tiff]

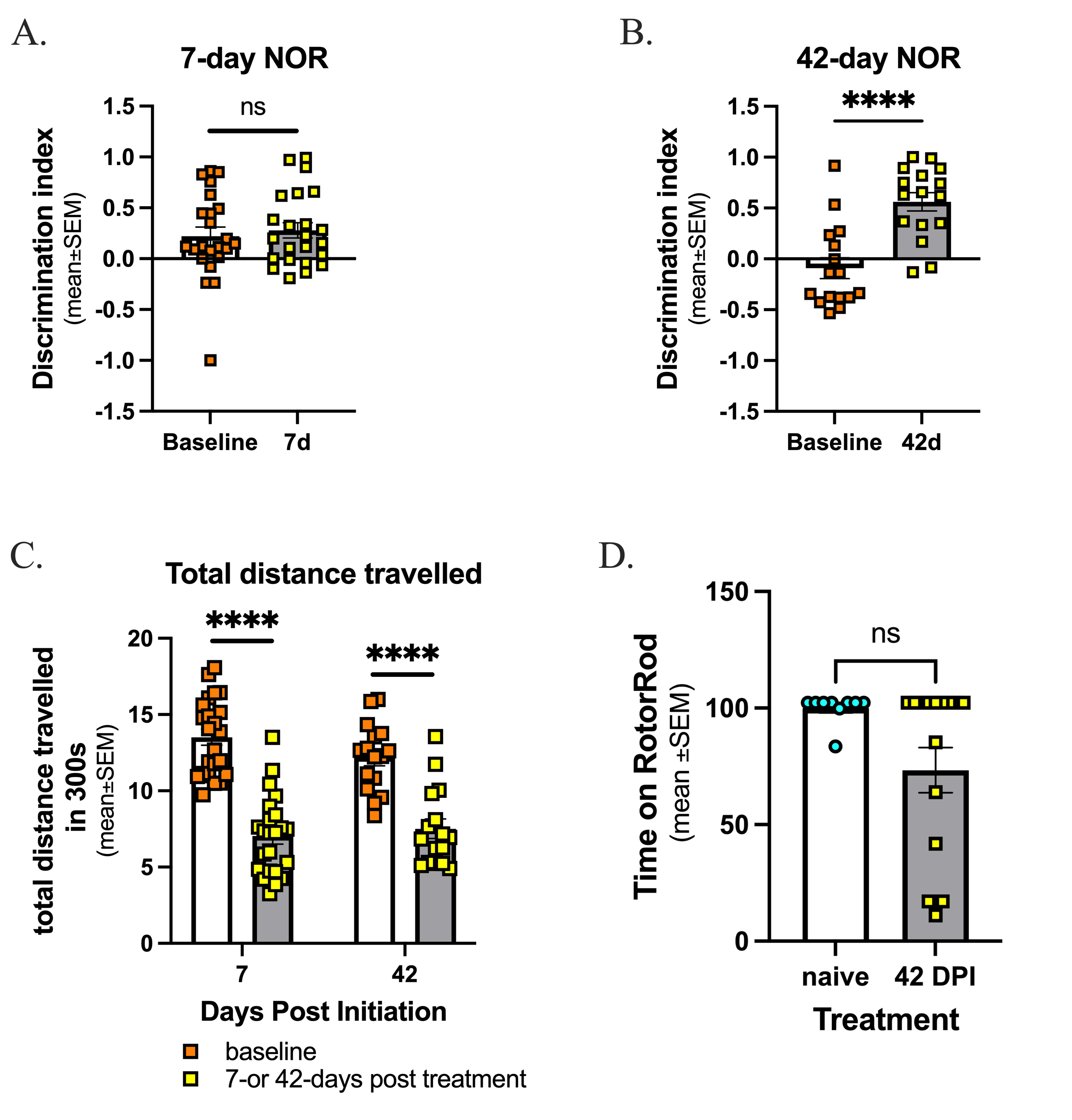

Supplement: Supplementary file 5 — Supplementary Material 5. [file 10194_2024_1907_MOESM5_ESM.tiff]
